# On flavonoid accumulation in different plant parts: variation patterns among individuals and populations in the shore campion (*Silene littorea*)

**DOI:** 10.3389/fpls.2015.00939

**Published:** 2015-10-29

**Authors:** José C. del Valle, Ma L. Buide, Inés Casimiro-Soriguer, Justen B. Whittall, Eduardo Narbona

**Affiliations:** ^1^Área de Botánica, Departamento de Biología Molecular e Ingeniería Bioquímica, Universidad Pablo de OlavideSeville, Spain; ^2^Department of Biology, College of Arts and Sciences, Santa Clara UniversitySanta Clara, CA, USA

**Keywords:** anthocyanins, Caryophyllaceae, coastal dune ecosystem, flavones, flavonols, flower color, plasticity, intra- and inter-population variation

## Abstract

The presence of anthocyanins in flowers and fruits is frequently attributed to attracting pollinators and dispersers. In vegetative organs, anthocyanins and other non-pigmented flavonoids such as flavones and flavonols may serve protective functions against UV radiation, cold, heat, drought, salinity, pathogens, and herbivores; thus, these compounds are usually produced as a plastic response to such stressors. Although, the independent accumulation of anthocyanins in reproductive and vegetative tissues is commonly postulated due to differential regulation, the accumulation of flavonoids within and among populations has never been thoroughly compared. Here, we investigated the shore campion (*Silene littorea*, Caryophyllaceae) which exhibits variation in anthocyanin accumulation in its floral and vegetative tissues. We examined the *in-situ* accumulation of flavonoids in floral (petals and calyxes) and vegetative organs (leaves) from 18 populations representing the species' geographic distribution. Each organ exhibited considerable variability in the content of anthocyanins and other flavonoids both within and among populations. In all organs, anthocyanin and other flavonoids were correlated. At the plant level, the flavonoid content in petals, calyxes, and leaves was not correlated in most of the populations. However, at the population level, the mean amount of anthocyanins in all organs was positively correlated, which suggests that the variable environmental conditions of populations may play a role in anthocyanin accumulation. These results are unexpected because the anthocyanins are usually constitutive in petals, yet contingent to environmental conditions in calyxes and leaves. Anthocyanin variation in petals may influence pollinator attraction and subsequent plant reproduction, yet the amount of anthocyanins may be a direct response to environmental factors. In populations on the west coast, a general pattern of increasing accumulation of flavonoids toward southern latitudes was observed in calyxes and leaves. This pattern corresponds to a gradual increase of UV-B radiation and temperature, and a decrease of rainfall toward the south. However, populations along the southern coast exposed to similar climatic stressors showed highly variable flavonoid contents, implying that other factors may play a role in flavonoid accumulation.

## Introduction

Flavonoids are secondary metabolites common to angiosperms, which confer a variety of biological functions (Gould and Lister, 2006; Agati et al., 2012). Anthocyanins, a group of flavonoids, are synthesized in the anthocyanin biosynthetic pathway (ABP), a highly conserved route of flavonoid biosynthesis. Branches of the ABP may lead to important groups of metabolites such as aurones, chalcones, flavones or flavonols (Davies and Schwinn, 2006; Saito et al., 2013). Anthocyanins show a variety of colors from blue to red, but their flavonoid intermediates are largely colorless, with the exception of aurones and chalcones that are yellow or pale- yellow (Tanaka et al., 2008). The accumulation of anthocyanins in flowers or fruits is commonly related to pollinator attraction and seed/fruit dispersers (Schaefer and Ruxton, 2011). However, in vegetative organs, anthocyanins and flavonols may perform a variety of functional roles in response to biotic and abiotic stressors such as UV radiation, cold, heat, drought, salinity, herbivory, pathogens, etc. (Chalker-Scott, 1999; Falcone Ferreyra et al., 2012; Narbona et al., 2014). In flowers, anthocyanin expression is generally constitutive and honed by the preferences of pollinators and the light environment (Fenster et al., 2004; Schiestl and Johnson, 2013). Conversely, in vegetative tissues, anthocyanins and other flavonoids usually accumulate transiently, as a plastic response to biotic or abiotic stressors (Manetas, 2006; Hatier and Gould, 2009).

Plants can differentially regulate anthocyanins in various tissues, organs and cell-types. Therefore, species with anthocyanin-pigmented flowers may or may not accumulate anthocyanins in vegetative tissues (e.g., Wheldale, 1916; Streisfeld and Kohn, 2005), and the same with anthocyanin accumulation in stems and leaves (e.g., Gould et al., 2010; Hughes et al., 2010). Their production can be variable among cells within the same tissue. This is the case of variegated flowers or flowers with striped petals (Schwinn et al., 2006; Shang et al., 2011) and of leaves with red margins (Albert et al., 2015). In white-flowered plants, the petal-specific downregulation of the ABP allows plants to avoid the negative effects of anthocyanin and flavonoid loss in vegetative organs (Strauss and Whittall, 2006; Streisfeld and Rausher, 2011). This tissue or cell-specific regulation is possible due to ABP gene regulation at the transcriptional level by the transcription factor MYB-bHLH-WD repeat (MBW) complex (Koes et al., 2005; Davies et al., 2012). Thus, the MBW complex allows plants to spatially and temporally change their anthocyanin production (Sobel and Streisfeld, 2013; Albert et al., 2014). In addition, it has been demonstrated that members of the MBW can be regulated by environmental conditions, such as light and temperature (Lu et al., 2009; Albert et al., 2011; Zoratti et al., 2014). Interestingly, some light-regulated transcription factors may control both anthocyanin production in vegetative tissues and in petals (Albert et al., 2011; Maier et al., 2013), indicating that anthocyanin accumulation in petals may not always be completely decoupled from vegetative tissues. This agrees with historic observations in which alpine and artic species show greater amount of anthocyanins at the whole plant level (Wheldale, 1916). Although the molecular basis of tissue or cell-specific regulation of anthocyanin production has been elucidated in several species (Albert et al., 2014), confirmation of the independent accumulation of anthocyanins and other flavonoids in different parts of plants in the field remains limited (but see Koski and Ashman, 2015).

Because most flavonoids are plastically produced as an acclimation process to environmental stressors (Manetas, 2006; Albert et al., 2011; Anderson et al., 2013; Hectors et al., 2014), individuals in different populations exposed to varying environmental conditions usually show variable accumulation of flavonoids (Jaakola and Hohtola, 2010). Environmental factors (temperature, precipitation, solar radiation, etc.) in populations throughout the species distribution area are often subjected to latitudinal, longitudinal, or altitudinal gradients (Narbona et al., 2010; Arista et al., 2013; Prendeville et al., 2013); thus, flavonoid accumulation may show geographic clines. For instance, flavonoid content in fruits of two species of *Vaccinium* showed a geographical gradient, with higher amounts of flavonoids in northern latitudes, probably due to the length of the day (Lätti et al., 2008, 2010). Flavonoid contents in *Betula pubescens* leaves were positively correlated with latitude (Stark et al., 2008). In European populations of *Plantago lanceolata*, latitude and altitude have a strong influence on the accumulation of anthocyanins in inflorescences, suggesting that these geographic effects are caused by the local thermal environment (Lacey et al., 2010). Recently, it has been demonstrated that plants of *Argentina anserina* showed an increased pattern of floral pigmentation (UV-absorbing flavonoids) in populations at lower latitudes in both hemispheres (Koski and Ashman, 2015), which confirms an analogous hypothesis for animals, of increased pigmentation toward equatorial latitudes (Lincoln et al., 1998). Thus, analyzing flavonoid accumulation in a variety of populations subjected to climatic gradients may be useful to identify potential environmental factors influencing anthocyanin production (Santamaría et al., 2003). Coastal species represent an ideal study system because the physical environment of their populations is very homogenous (elevation, topography, etc.), yet there is a wide range of climatic conditions among populations along a latitudinal gradient (Sagarin et al., 2006).

**Figure 1:**
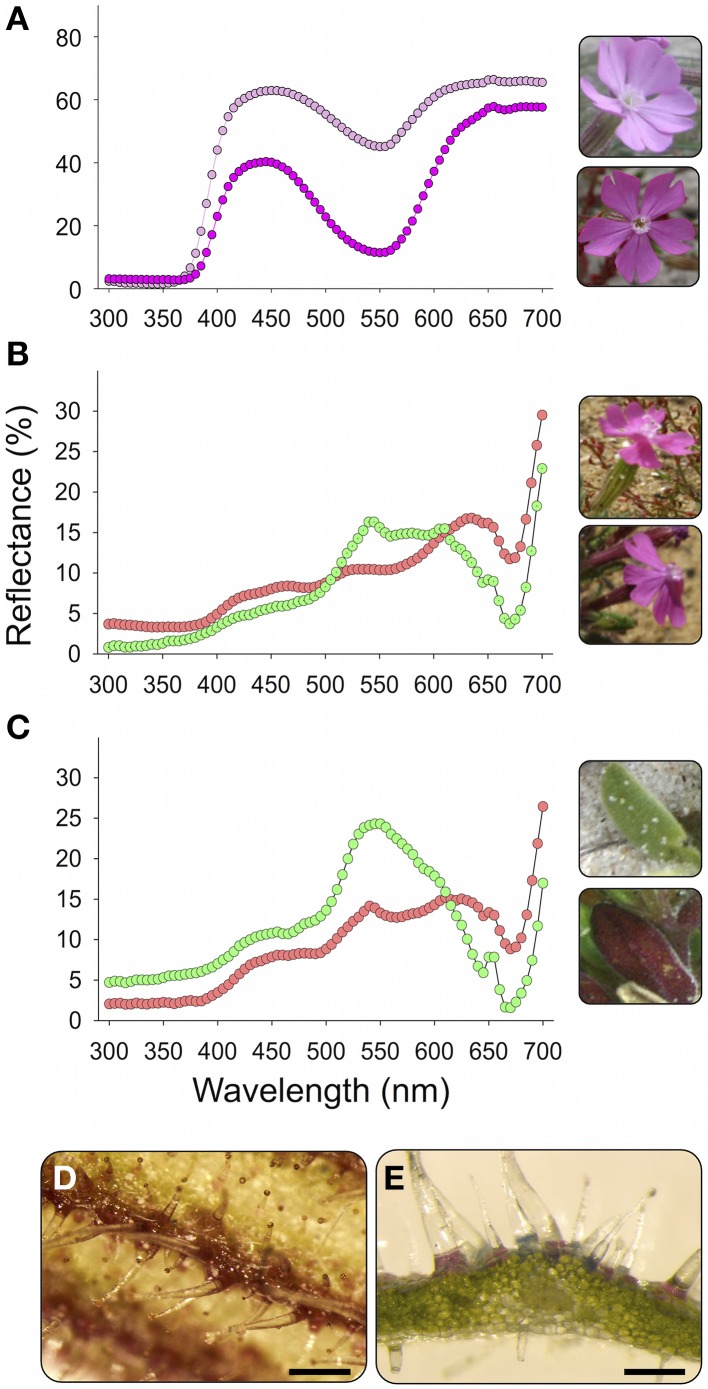
**Petals (A), calyxes (B), and leaves (C) of *Silene littorea* and their UV-Vis reflectance spectra**. Two contrasting populations in redness were chosen for each plant organ: Alc (dark pink circles) and Are (light pink) for petals, Sin (red circles) and Bre (green circles) for calyxes, and San (red circles) and Tra (green circles) for leaves (see Table 1 for population code). The mean population reflectance spectrum is showed. For details of spectra acquisition see Buide et al. (2015). **(D)** Detail of a calyx showing superficial accumulation of anthocyanins in the veins, scale = 0.5 nm. **(E)** Detail of a transversal section of a leaf showing accumulation of anthocyanins in epidermal cells of abaxial surface exposed to light, scale = 0.2 nm.

In this study, we investigated the accumulation of anthocyanins and other flavonoids in reproductive (petal and calyx) and vegetative (leaf) organs of *Silene littorea* Brot. (Caryophyllaceae) in 18 populations across their geographic range. This entomophilous pink-flowered species has calyxes, stems and leaves that range from light green to dark red depending on the amount of anthocyanin produced (Figure 1). This pigmentation is caused by the accumulation of anthocyanins (cyanidin-3-glucoside derivatives), but other flavonoids such as flavones and flavonols are also present in vegetative and reproductive tissues (Casimiro-Soriguer, 2015). Although betalains are produced in some families of the Caryophyllales, only anthocyanins are documented in the Caryophyllaceae (Brockington et al., 2011). The redness of vegetative tissues is a highly variable character (Narbona et al., 2014), which may be a plastic response to UV-B light (Del Valle et al., unpublished data). Gene expression analyses suggest that MYB transcription factors could be involved in natural within-population variation of petal color intensity (Casimiro-Soriguer, 2015). *S. littorea* specifically inhabits foredunes from the northwest (43°N, 8°W) to the southeast (36°N, 1°W) of the Iberian Peninsula. Thus, populations are exposed to a high degree of solar exposure, temperature and precipitation (Supplementary Table 1). In contrast, within a given population, environmental conditions are very homogeneous, and the same is true for several among-population conditions such as soil properties and vegetation composition (Lomba et al., 2008).

Within this framework, we predicted how anthocyanin and non-anthocyanin flavonoid accumulation varies among different organs within a single plant, and among individuals or populations across the species geographic range. Considering that the biosynthesis of flavonoids can be tissue-specifically regulated (e.g., Davies et al., 2012) and also represent a cost for the plant (Steyn et al., 2002), we predict that flavonoid content in different organs of the plants will not be correlated. In addition, because flower color is subject to pollinator-mediated selection (e.g., Fenster et al., 2004), we expect that the amount of anthocyanins in the petals will vary to a lesser extent than those in the calyxes and leaves. On the other hand, anthocyanins and other flavonoids that accumulate in the same tissue are generally positively related, because they are synthesized in the general flavonoid biosynthetic pathway, and the same transcription factor may simultaneously regulate several steps in the pathway (Davies and Schwinn, 2006, but see Mouradov and Spangenberg, 2014). Consequently, we also predict that anthocyanins and non-anthocyanin flavonoids will be correlated within each specific organ. Finally, considering the protective role of flavonoids (Agati et al., 2012; Falcone Ferreyra et al., 2012) and plastic accumulation due to climatic conditions (Lacey et al., 2010), we hypothesize that flavonoids will accumulate to different levels across the geographic range of *S. littorea* in relation to climate, specifically we expect a positive correlation with temperature and UV-B radiation and a negative correlation with precipitation (Chalker-Scott, 1999; Koski and Ashman, 2015). Thus, we address the following questions: How do the flavonoid contents vary in different plant organs (petals, calyxes, and leaves) within and among populations? Is there a relationship between anthocyanin and non-anthocyanin flavonoids that accumulate in each plant organ? Do the flavonoid contents of each plant organ show a geographical pattern related to the climate features of the populations?

## Materials and methods

### Study system and sampling

*Silene littorea* is a self-compatible species mainly pollinated by butterflies, bees and moths (Del Valle, unpublished data). This annual plant blooms between March and June and exhibits large variation in flower production, from three to ca. 300 flowers per plant (mean ± s.e. = 97.4 ± 9.1, Casimiro-Soriguer et al., 2013). Most *S. littorea* plants have pink petals with a range of intensity across populations (Figure 1A). In two populations from northwestern Spain (Bar and Lou, Supplementary Table 1) white petals can be found on ca. 20% of plants (Narbona et al., 2014). In these two polymorphic populations, white-flowered individuals were not included in this study. Anthocyanin accumulation in calyxes and leaves produces a continuous variety of colored organs from light green to dark red (Figures 1B,C). Within an individual plant, the color of each tissue is homogeneous; thus, we only sampled once per plant. The flavonoid derivatives present in *S. littorea*, identified by HPLC-ESI-MS/MS, were the flavones apigenin, isovitexin and luteolin; the flavonols rutin and quercetin; and the anthocyanin cyanidin-3-glucoside (Casimiro-Soriguer, 2015). In both calyxes and leaves, anthocyanins are accumulated in epidermal cells (Figures 1D,E).

**Figure 2:**
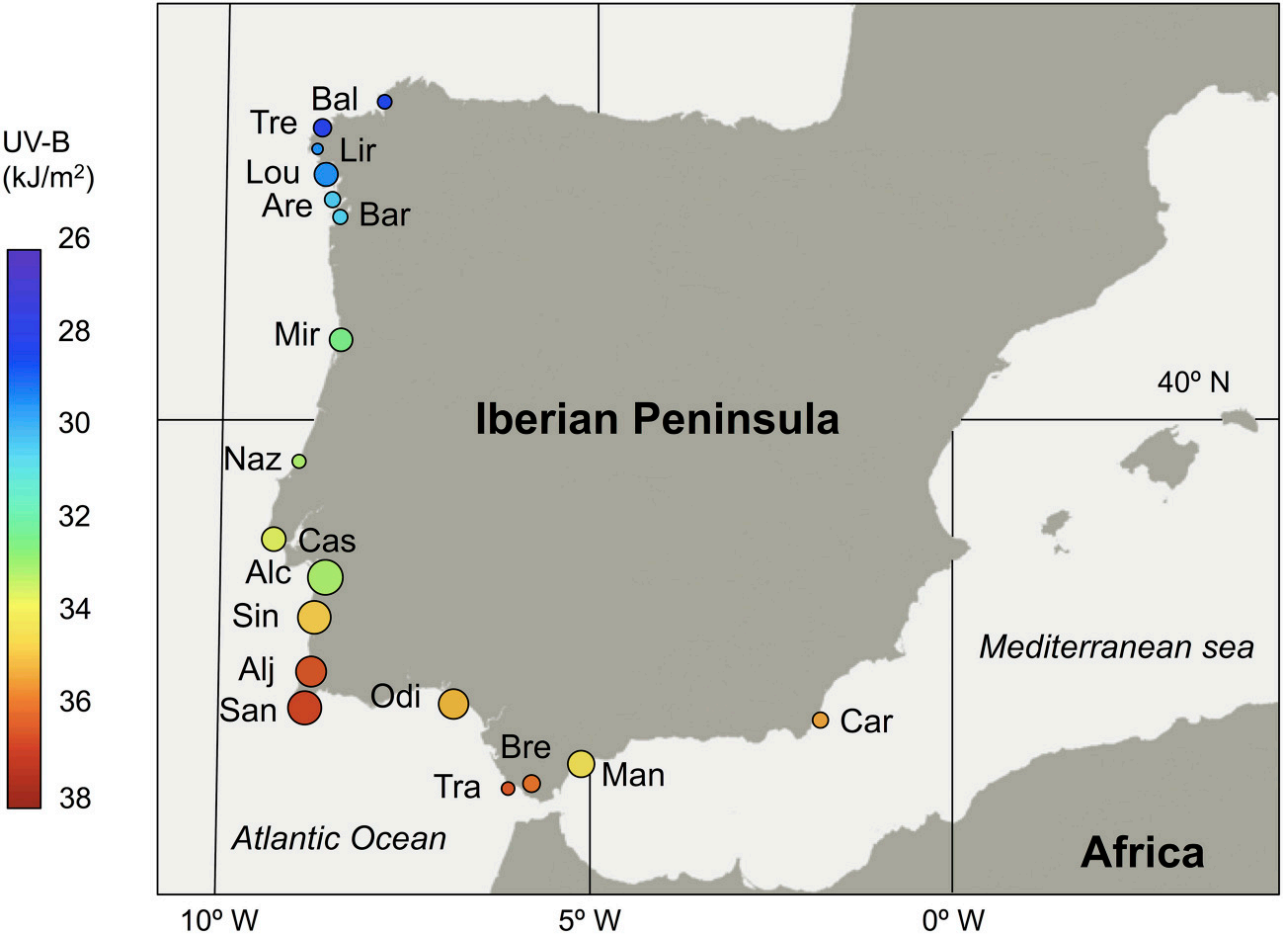
**Geographical distribution of studied populations of *S. littorea* in the Iberian Peninsula**. Population mean anthocyanin contents in the calyxes were represented by means of the relative point size (original values are showed in Table 1). Incident UV-B radiation (kJ/m^2^) on each population is represented by means of a color scale inside the circle. Population code is showed in Supplementary Table 1.

Samples were collected from 2012 to 2014 during the peak-flowering period. We have observed several populations during the flowering season in three consecutive years, and we did not detect any color change within individuals, nor among the entire population for floral and vegetative tissues. We randomly sampled 15–31 individuals per population for 18 populations from the northwestern to southeastern portions of the Iberian Peninsula (Figure 2; Supplementary Table 1).

### Flavonoids quantification

In order to quantify the *in-situ* accumulation of flavonoids, a branch or a whole plant was placed in zip-lock bag and transported inside a cooler with ice until later extraction (usually in within 6 h with no apparent color change). For each individual, four petals and the calyx of one flower were dissected. In addition, a section of approximately 1 centimeter of length of a leaf from the middle region of the stem was selected. Weights of calyxes and leaves were measured using a precision scale. The petals were photographed on graph paper and the total area was measured using the software ImageJ v.1.48v (National Institutes of Health, USA); the petal's weight was calculated using the mean weight per area (2.11 μg cm^−2^, *N* = 8). Samples were preserved in 1 mL of CH_3_OH:H_2_0 (7:3, v:v) containing 1% HCl and stored at −20° C in the dark until the subsequent pigment extraction. Petals were homogenized using 3 mm diameter glass beads (EMD Millipore Corporation, Billerica, MA), and beaten in a bead beater for 1 min intervals until samples were thoroughly homogenized. The supernatant was removed after 10 min centrifugation (13,000 rpm) and stored at −80°C. The same procedure was applied for calyx and leaf samples, but these plant parts were previously frozen in liquid nitrogen to improve homogenization.

Three replicates of 200 μL per flavonoid extraction were placed in a Multiskan GO microplate spectrophotometer (Thermo Fisher Scientific Inc., MA, USA). Extracts of each plant part were scanned in order to identify the absorption maximum of the different flavonoids, using the wavelengths commonly used in the literature to determine the concentration of the main groups of flavonoids (Merken and Beecher, 2000; Shimada et al., 2005; Zhu et al., 2012); absorbances were read at 520 nm for anthocyanins and at 350 nm for combined non-anthocyanin flavonoids (flavones and flavonols). We estimated combined non-anthocyanin flavonoids content at 350 nm because the amount of flavones (absorbance peak at 350 nm) in samples of *S. littorea* is 2.5 times higher than that of flavonols (peak at 370 nm; Casimiro-Soriguer, 2015). In calyx and leaf samples, anthocyanin content was corrected using the formula A_520_–0.24 × A_653_ in order to compensate for the small overlap in absorption by chlorophyll (Murray and Hackett, 1991; Gould et al., 2000). Anthocyanin and non-anthocyanin flavonoid content were quantified using five-point calibration curves of cyanidin-3-glucoside chloride and luteolin standards (Sigma-Aldrich, Steinheim, Germany) and expressed as cyanidin-3-glucoside and luteolin equivalents, respectively.

### Relationship between amounts of anthocyanins and climatic features

Mean temperature and cumulative rainfall were extracted from the Digital Climatic Atlas of Spain (Ninyerola et al., 2005) using 15 and 50 year datasets for local weather stations. Total solar and UV-B (280–315 nm) radiation values were obtained from the Solar Radiation Data (SoDa Services), an online data service that provides daily mean surface solar irradiance for the period 1985–2005 from the HelioClim-1 database. This database has been created from archives of images of the Meteosat satellites using the Heliosat-2 method (Rigollier et al., 2004). Solar and UV-B radiation was highly correlated; thus, we used UV-B radiation in our analyses due to the known functional relationship between flavonoids and UV-B (Jansen et al., 1998; Hectors et al., 2014). Climate data was restricted to the growing period of *S. littorea* (February–May; Supplementary Table 1).

### Statistical analysis

For each plant organ, the variation of the flavonoid content among populations was explored with a linear mixed-effects model, considering the “population” as a random factor (Bennington and Thayne, 1994). In these analyses, the components of variance for the population factor and the error term (plant nested within population) were estimated (Crawley, 2007). The inter- and intra-population correlations of anthocyanin and non-anthocyanin flavonoid contents among petals, calyxes, and leaves were assessed with Pearson correlations and a Bonferroni adjustment for multiple comparisons (Rice, 1989). A similar analysis was carried out to test possible relationships between the anthocyanin and non-anthocyanin flavonoid contents in each plant organ.

We also evaluated whether flavonoid contents in each plant organ were related to the main climatic variables of the populations (UV-B radiation, temperature and precipitation). Although the multicollinearity of these environmental variables and latitude (see Results) make the use of multivariate statistics unsuitable (Kutner et al., 2005; Grace, 2006), we performed a principal component analysis (PCA) to explore the relative importance of all climatic and geographic variables. In each organ, linear regressions with Bonferroni adjustments were performed between latitude (see Results) and the mean population flavonoid contents. In addition, to test if flavonoid contents were spatially autocorrelated (i.e., similar contents values in adjacent populations), Mantel tests with pairwise geographic distances based on latitude and longitude were carried out using the “ade4” R package (Crawley, 2007).

Data were Box-Cox transformed to meet normality and homoscedasticity assumptions. The normality was tested with the Shapiro-test and homoscedasticity was checked with the Fligner–Killeen statistic (Crawley, 2007). All analyses were performed in R version 3.1.1 (R Core Team, 2013), with the exception of PCA that was performed in Tanagra software (http://eric.univ-lyon2.fr/~ricco/tanagra/en/tanagra.html).

## Results

### Variation in flavonoid contents among plants and populations

Considering the whole data set, the average content of anthocyanins in petals, calyxes, and leaves were 28.18 ± 1.49 mg g^−1^ fresh weight (hereafter FW; mean ± SE), 2.72 ± 0.14 mg g^−1^ FW, and 0.75 ± 1.00 mg g^−1^ FW, respectively. The average non-anthocyanin flavonoid content in each organ was much higher compared to anthocyanins: 127.27 ± 7.01 mg g^−1^ FW, 16.31 ± 0.90 mg g^−1^ FW, and 16.05 ± 0.88 mg g^−1^ FW for petals, calyxes and leaves, respectively. This relationship was consistent among populations (Table 1).

**Table 1:** **Descriptive statistics of anthocyanin and non-anthocyanin flavonoid contents (mg g^−1^ FW) in petals, calyxes, and leaves of *S. littorea* populations (ordered from NW to SE)**.

**Pop**.	**Anthocyanins^a^**						**Non-anthocyanin flavonoids^b^**					
	**Petals**		**Calyxes**		**Leaves**		**Petals**		**Calyxes**		**Leaves**	
	Mean±SE	CV	Mean±SE	CV	Mean±SE	CV	Mean±SE	CV	Mean±SE	CV	Mean±SE	CV
Bal	13.0±1.0	35.4	1.75±0.14	37.7	0.38±0.09	107.7	90.1±4.5	22.9	10.9±0.49	20.3	11.9±0.94	36.3
Tre	46.3±2.1	20.9	2.17±0.16	33.9	0.14±0.03	80.4	218.3±8.7	17.8	13.8±1.61	38.8	15.7±0.79	18.7
Lir	11.7±0.9	36.2	2.19±0.16	34.0	0.39±0.04	44.1	79.1±2.5	14.5	8.7±0.33	17.2	9.5±0.66	32.0
Lou	20.9±2.7	47.8	3.52±0.44	46.5	0.38±0.05	47.1	169.3±28.0	40.6	18.0±1.24	24.8	13.8±0.74	19.3
Are	6.7±0.4	27.5	2.92±0.26	41.2	0.62±0.10	74.9	73.4±2.3	14.5	12.2±0.78	29.4	11.6±0.55	21.7
Bar	19.7±1.5	38.4	2.19±0.24	55.0	0.33±0.04	65.7	137.2±4.2	14.6	11.1±0.55	24.9	16.1±0.95	30.1
Mir	27.5±1.6	27.8	2.49±0.35	65.2	0.40±0.05	50.0	186.6±14.8	23.8	17.8±1.53	35.4	14.8±1.08	33.4
Naz	40.1±2.4	27.7	1.86±0.23	57.7	0.56±0.06	49.0	154.7±7.8	23.0	10.4±0.51	18.9	12.6±0.66	23.5
Cas	46.0±3.6	35.6	2.93±0.36	50.7	0.41±0.07	78.5	129.7±5.1	18.1	18.4±1.31	32.6	12.0±1.14	43.5
Alc	55.7±5.7	43.0	4.66±0.52	48.1	2.21±0.48	83.8	148.1±9.1	26.1	26.9±2.46	38.9	24.5±2.38	40.1
Sin	23.2±2.3	42.2	4.61±0.52	46.2	1.35±0.40	115.3	145.9±10.8	28.6	25.4±1.86	30.3	18.5±2.17	45.4
Alj	38.7±1.3	14.5	3.99±0.37	41.7	1.76±0.30	76.7	103.2±4.6	20.0	23.4±1.63	31.1	25.9±2.68	45.0
San	56.5±3.6	26.7	5.91±0.41	29.8	2.16±0.29	56.8	142.5±7.5	22.3	25.7±1.91	30.6	16.8±1.31	31.0
Odi	35.0±1.5	19.5	3.69±0.48	58.2	1.82±0.28	65.7	133.5±5.6	17.9	23.2±1.59	27.4	33.5±2.50	29.8
Tra	15.1±1.2	43.7	1.05±0.12	64.0	0.11±0.03	156.7	122.2±3.1	14.1	10.0±0.63	33.1	10.0±0.55	27.5
Bre	23.6±1.3	19.7	1.11±0.14	46.5	0.34±0.06	56.9	93.7±4.2	15.6	13.1±0.77	21.1	22.2±2.29	32.6
Man	18.6±2.4	50.2	1.60±0.17	43.0	0.49±0.11	93.8	159.3±9.7	22.7	20.5±1.06	20.8	16.0±0.77	19.9
Car	17.4±1.0	27.3	1.97±0.21	49.4	0.53±0.05	44.7	80.4±3.3	18.7	11.9±0.69	26.4	14.1±0.89	29.1
Total	28.2±1.5	63.5	2.72±0.14	68.2	0.75±0.04	133.0	127.3±7.0	36.6	16.3±0.90	49.2	16.0±0.88	51.2

a *Determined as cyanidin-3-glucoside equivalents*.

b *Determined as luteolin equivalents. SE, standard error; CV, coefficient of variation expressed in percentage. Population code is show in Supplementary Table 1*.

The contents of anthocyanins and non-anthocyanin flavonoids in each organ of *S. littorea* varied within and among populations (Table 1). The coefficients of variation (CV) of anthocyanin content in the whole dataset were approximately twice as high in leaves (133.0%) as in petals and calyxes (63.5 and 68.2%, respectively). However, the non-anthocyanin flavonoid content was less variable, with overall CV of 36.6% in petals and ca. 50% in calyxes and leaves.

Differences in anthocyanin content among populations were statistically significant for petals [*t*_(17, 340)_ = 26.97, *P* < 0.0001], calyxes [*t*_(17, 342)_ = 34.29, *P* < 0.0001] and leaves [*t*_(17, 299)_ = 64.71, *P* < 0.0001]. In petals, the proportion of anthocyanins content variation among populations was three times higher than within population variation (73.37 vs. 26.63%). However, the within population and among population variance was nearly similar in both calyxes (47.51 vs. 52.49%, respectively) and leaves (47.75 vs. 52.25%). We also found significant differences among populations in the non-anthocyanin flavonoids for the petals [*t*_(17, 312)_ = 68.54, *P* < 0.0001], calyxes [*t*_(17, 312)_ = 78.23, *P* < 0.0001], and leaves [*t*_(17, 316)_ = 77.53, *P* < 0.0001]. In petals, there was higher variance among populations than within populations (71.33 vs. 28.67%). Yet, in calyxes there was higher variance within populations than among, but to a lesser degree (56.98 vs. 43.02%). The variance for leaf anthocyanin content was similar for within vs. among population comparisons (48.53 vs. 51.47%).

### Relationships between the flavonoid contents in different organs

**Figure 3:**
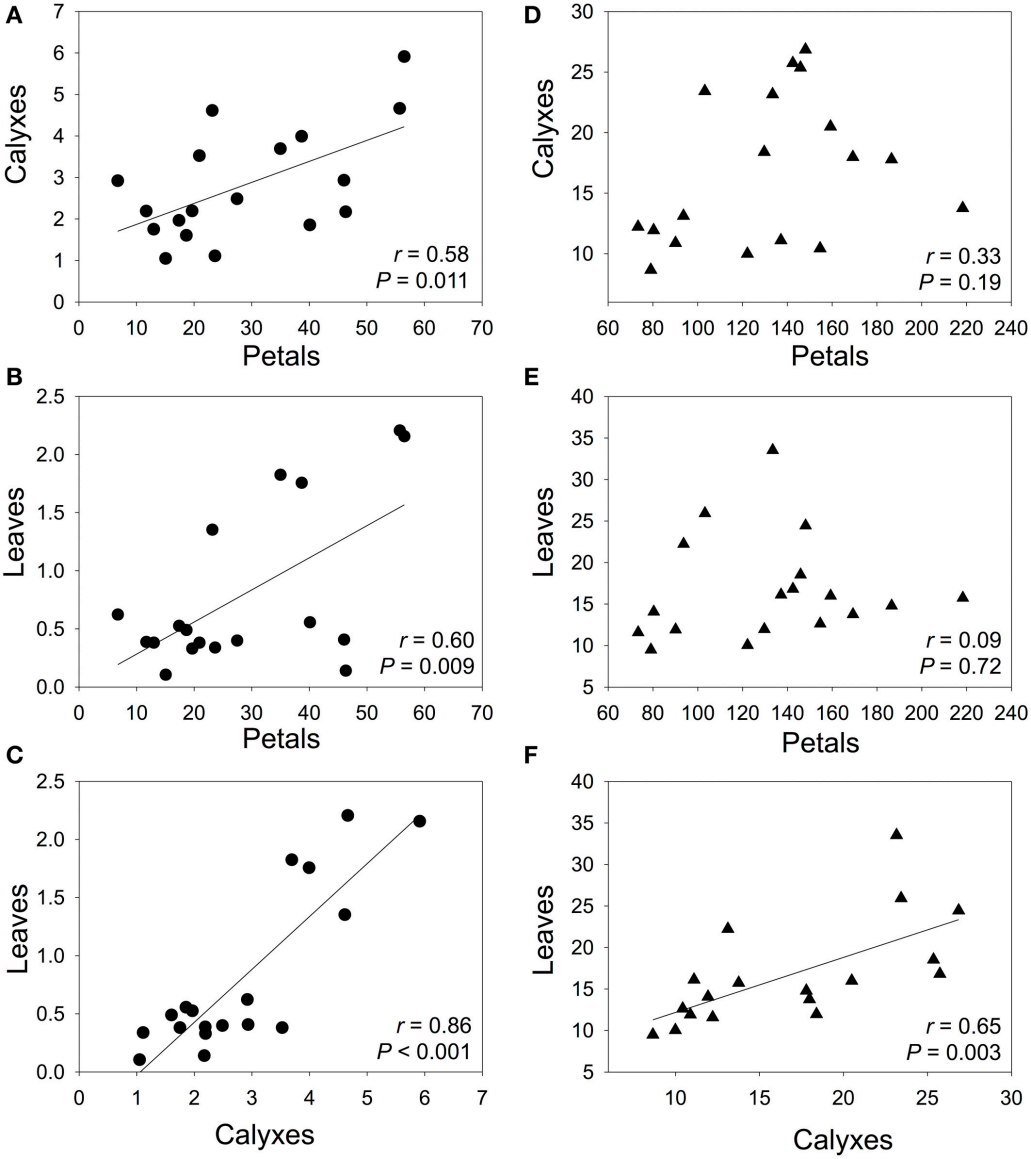
**Pearson correlations between population mean content of flavonoids in petals vs. calyxes (A, B), petals vs. leaves (C, D), and calyxes vs. leaves (E, F)**. Circles and triangles represent the content (mg g^−1^ FW) of anthocyanins and non-anthocyanin flavonoids, respectively. The best-fit lines were showed for significant correlations. *P*-values are significant at Bonferroni-corrected *P* level (0.05/3 = 0.017).

In general, neither anthocyanin content, nor non-anthocyanin flavonoid content correlated among petals, calyxes, and leaves within each population (Table 2). However, when we analyzed the average values of each population, we found that the population mean anthocyanin content between different plant organs were significantly correlated; the correlation coefficient was high in calyxes vs. leaves but moderate in petals vs. calyxes and in petals vs. leaves comparisons (Figures 3A–C). For the non-anthocyanin flavonoids, a significant correlation was only present in calyxes vs. leaves (Figures 3D–F).

**Table 2:** **Pearson correlation coefficients comparing anthocyanin and non-anthocyanin flavonoid contents in petals, calyxes, and leaves of *S. littorea* populations**.

**Anthocyanins**				**Non-anthocyanin flavonoids**		
**Population**	**Petal vs. Calyx**	**Petal vs. Leaf**	**Calyx vs. Leaf**	**Petal vs. Calyx**	**Petal vs. Leaf**	**Calyx vs. Leaf**
Bal	−0.05	0.09	0.48	0.01	0.01	0.43
Tre	0.34	−0.12	−0.20	−0.67	−0.13	−0.03
Lir	0.22	−0.20	−0.15	0.43	−0.03	0.01
Lou	0.68^*^	0.06	−0.11	0.82	0.22	0.09
Are	0.10	0.06	0.50	−0.59^*^	−0.15	0.26
Bar	0.10	0.27	−0.04	−0.27	−0.16	0.25
Mir	0.11	0.33	0.32	−0.69	−0.18	0.64^*^
Naz	−0.19	0.05	0.34	−0.31	0.23	−0.20
Cas	0.11	−0.05	0.23	0.35	−0.06	−0.36
Alc	0.00	−0.16	−0.31	−0.02	−0.01	−0.31
Sin	−0.04	−0.02	0.63	−0.07	0.17	0.54
Alj	−0.16	0.33	0.11	0.07	−0.11	0.24
San	−0.17	−0.37	0.32	0.27	−0.04	0.60
Odi	0.30	0.15	0.36	−0.10	−0.31	−0.22
Tra	0.28	0.07	0.39	0.24	0.10	−0.59^*^
Bre	−0.01	0.42	0.66	−0.16	0.21	0.68
Man	0.27	−0.08	0.46	−0.22	0.36	0.02
Car	0.05	−0.21	−0.50	0.03	0.23	0.67^*^

* *P-values in bold are significant at Bonferroni-corrected P level (0.05/3 = 0.017)*.

### Relationship between anthocyanins and non-anthocyanin flavonoids in the same organ

**Figure 4:**
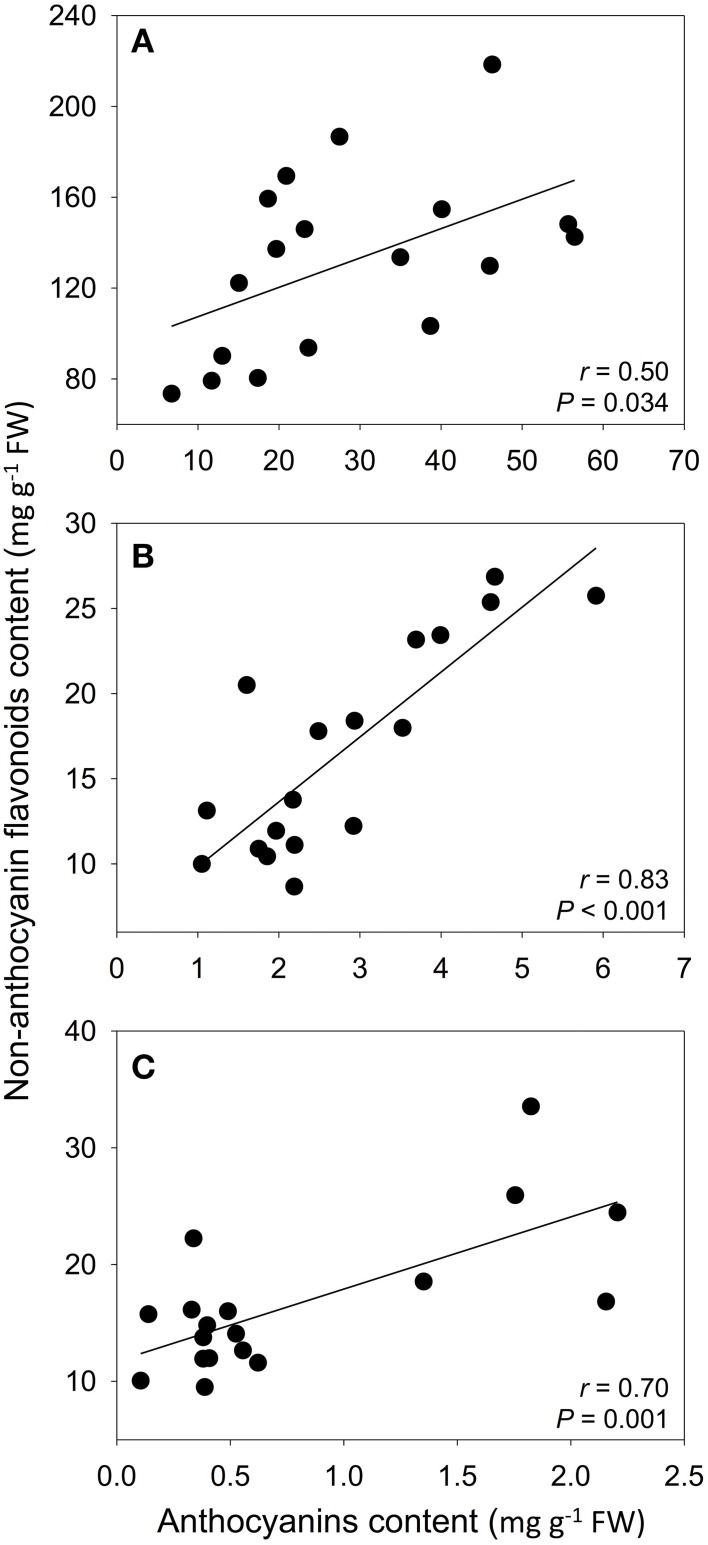
**Pearson correlations between the population mean content of anthocyanins and non-anthocyanin flavonoids in petals (A), calyxes (B), and leaves (C)**. Anthocyanins were determined as cyanidin-3-glucoside equivalents and non-anthocyanin flavonoids were determined as luteolin equivalents. The best-fit lines were drawn for significant correlations.

In petals and leaves, correlations between anthocyanins and non-anthocyanin flavonoid content were found in 10 and 6 populations, respectively. In calyxes, this correlation was found within almost all populations (Table 3). Considering the average values of all populations, the strongest relationship was between the calyxes and leaves (Figure 4).

**Table 3:** **Pearson correlation coefficients of the comparison between anthocyanins and non-anthocyanin flavonoid contents in each plant organ of *S. littorea* populations**.

**Population**	**Petals**	**Calyxes**	**Leaves**
Bal	0.56^*^	−0.38	0.05
Tre	0.93^***^	−0.74^*^	−0.16
Lir	0.44^*^	−0.58^*^	−0.68^*^
Lou	0.92^*^	0.69^*^	−0.38
Are	0.14	−0.59^*^	−0.37
Bar	0.48^*^	−0.52^*^	0.20
Mir	0.65	0.81^***^	0.36
Naz	−0.41	−0.61^*^	0.00
Cas	0.68^*^	0.62^*^	−0.59^*^
Alc	0.53^*^	0.77^*^	0.43
Sin	−0.21	0.59^*^	0.40
Alj	−0.35	0.79^***^	0.74^***^
San	**0.52**	0.88^***^	0.51^*^
Odi	0.31	0.52^*^	0.50
Tra	0.37^*^	0.75^***^	−0.13
Bre	0.52^*^	0.74^*^	0.76^*^
Man	−0.03	−0.66^*^	0.21
Car	0.26	−0.70^*^	0.81^***^

*Significant correlations were highlighted in bold*.

* P < 0.05;

*** *P < 0.0001*.

### Relationship between flavonoid contents and geographic and climatic factors

All climatic variables were strongly correlated (*r* > 0.88; Table 4). Latitude showed a strong correlation with all the climatic variables of *S. littorea* populations (*r* > 0.93; Table 4). Temperature and UV-B radiation were greater at lower latitudes, whereas precipitation had the opposite trend. In contrast, there was no correlation between longitude and climatic factors (Table 4). The PCA showed that the PC1 and PC2 explained 83.4 and 12.4% of the variance, respectively. In PC1, the three climatic variables and latitude showed high and similar factor loadings (ranged between 0.94 and 0.98), whereas longitude showed a loading of 0.69 (Supplementary Table 2). Given the similar loading of all the climatic factors and the latitude in PC1, as well as the high correlation among them, we used latitude as a representative variable for this group of variables.

**Table 4:** **Pearson correlation coefficients between climatic and geographic variables from the studied populations of *S. littorea***.

	**UV-B radiation**	**Mean temperature**	**Cumulative precipitation**
Mean temperature	0.88^***^		
Cumulative precipitation	−0.90^***^	−0.90^***^	
Latitude	−0.96^***^	−0.93^***^	0.93^***^
Longitude	−0.40	−0.28	0.37

*For climatic data calculations, the period from February to May were used. Signifcant correlations were highlighted in bold*.

*** *P < 0.0001*.

**Figure 5:**
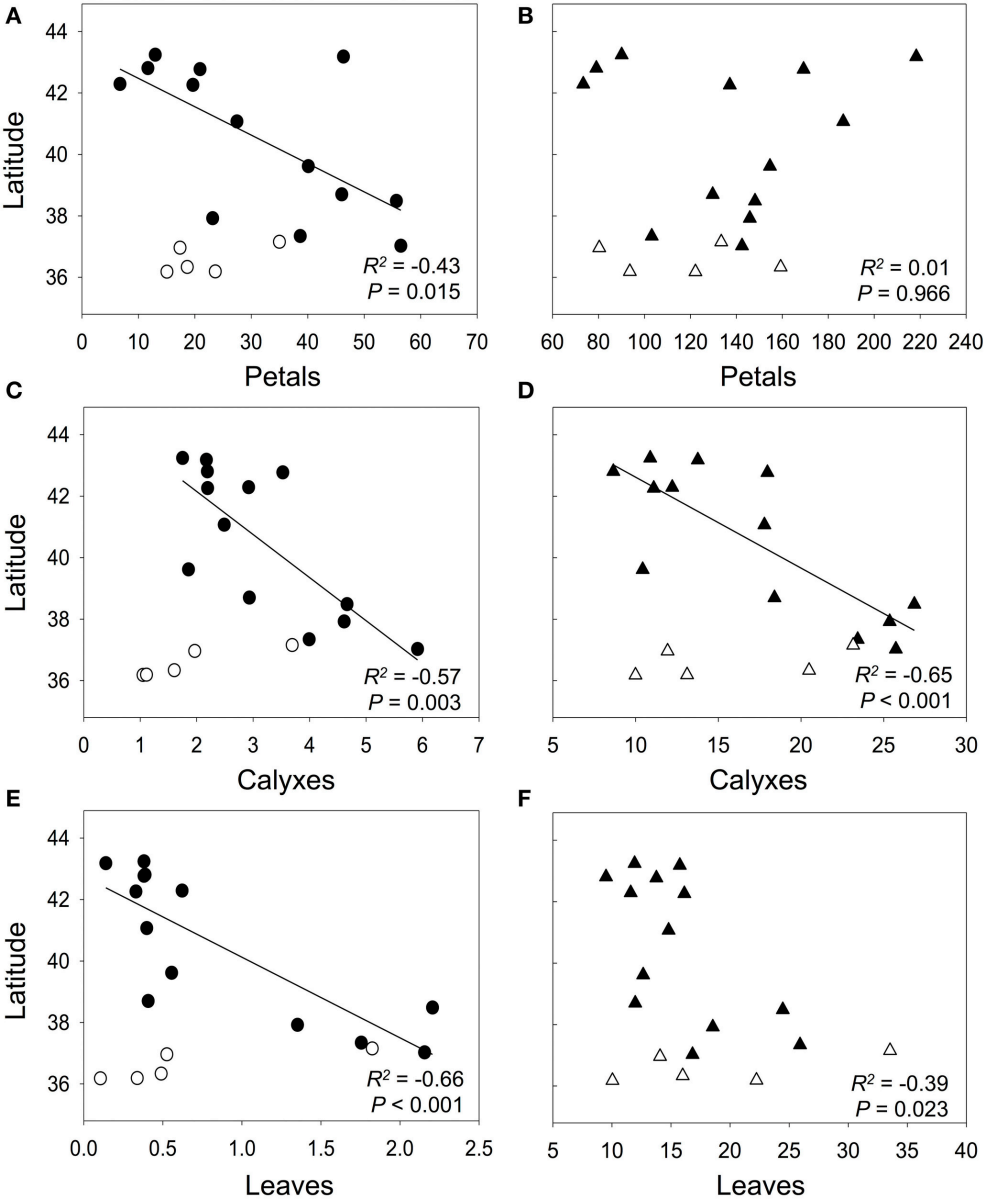
**Relationship between latitude and contents of anthocyanins (circles) and non-anthocyanin flavonoids (triangles) in petals (A,B), calyxes (C,D), and leaves (E,F)**. Black points represent populations from the west coast, whereas the white points represent populations of the south coast (i.e., Odi, Tra, Bre, Man, and Car populations; see Figure 2). Flavonoid contents are expressed as mg g^−1^ FW. *R*^2^ and the best-fit lines correspond to regression analysis of populations from the west coast. *P*-values are significant at Bonferroni-corrected *P* level (0.05/3 = 0.017).

Using all populations, we did not find a relationship between the anthocyanin contents and latitude in any plant organ (*R*^2^ = 0.06, *P* = 0.317 for petals; *R*^2^ = 0.02, *P* = 0.622 for calyxes; *R*^2^ = 0.17, *P* = 0.090 for leaves). Similar results were found for the non-anthocyanin flavonoids in petals (*R*^2^ = 0.02, *P* = 0.594), but in this case, calyxes and leaves showed marginally significant relationships (*R*^2^ = 0.19, *P* = 0.068; *R*^2^ = 0.19, *P* = 0.067, respectively). The Mantel tests showed absence of spatial autocorrelation for all plant organs and flavonoids types, except for the non-anthocyanin flavonoids in petals (*P* = 0.002; Supplementary Table 3). Conversely, if we consider only the populations on the west coast of the distribution area (i.e., populations from Bal to San, Figure 2), we observe a general pattern of increasing flavonoid contents toward southern latitudes (Figure 5). In anthocyanins of calyxes and leaves the relationship was highly significant but in petals, it was slightly significant (Figures 5A,C,E). In non-anthocyanin flavonoids, the relationship was highly significant in calyxes and marginally significant in leaves, whereas in petals the relationship was not significant (Figures 5B,D,F). The Mantel tests showed a significant autocorrelation in the flavonoid contents among population (*P* < 0.002 for the three plant organs; Supplementary Table 3).

## Discussion

### Patterns of flavonoid content variation within and among populations

Our results show that in each plant organ, the content of anthocyanins and other flavonoids varies greatly within and among populations. As expected, CVs of anthocyanin content in petals were much lower than in leaves, both at the population and the species level. Changes in the presence or absence of anthocyanins (i.e., pigmented vs. unpigmented flowers) may cause important changes in pollinator fauna due to pollinator preferences for particular colors (Fenster et al., 2004; Hoballah et al., 2007). Furthermore, changes in anthocyanin concentration may also affect petal color (i.e., hue and chroma, Holton et al., 1993; Schmitzer et al., 2009) and subsequent pollinator activity (Shang et al., 2011). Thus, the low variation in anthocyanin contents in petals of *S. littorea* suggests petal specific regulation and possibly some evolutionary constraint on that character because of the importance for pollinators (Schiestl and Johnson, 2013). With respect to non-anthocyanin flavonoid content, the CV of the petals was smaller than those of calyxes and leaves, which may be explained by the relationship between the contents of anthocyanins and other flavonoids in each organ (see below).

We found that both at the population and species levels, the anthocyanin content in calyxes was approximately four times higher than in leaves, but that of non-anthocyanin flavonoids was nearly similar in both plant parts. The content of non-anthocyanin flavonoids was 6 and 21 times higher than those of anthocyanins in calyxes and leaves, respectively. In petals, non-anthocyanin flavonoids were also five-fold more abundant than anthocyanins. This much higher content of non-anthocyanin flavonoids is common in plants (Jaakola et al., 2004; Zhu et al., 2012; Chen et al., 2013). Although, both groups of compounds show protective functions against biotic and abiotic stressors (Gould and Lister, 2006; Agati et al., 2012), the predominance of non-anthocyanin flavonoids suggests that by mass alone, the visible anthocyanins represent the minority of the flavonoids (yet the visible anthocyanins are often the focus of functional studies; Manetas, 2006).

### Anthocyanin and other flavonoid variations in the same and different organs

Within populations, there was no correlation between the flavonoid content in petals vs. calyxes, petals vs. leaves, and calyxes vs. leaves. These results suggest that in each plant, flavonoid production is regulated independently in each organ. In the white flowered *S. latifolia*, similar independent accumulation of anthocyanins and flavones in leaves and calyxes has been proposed (Mastenbroek and Van Brederode, 1986). Although, the independent accumulation of anthocyanins or other flavonoids in different plant parts has previously been documented as a qualitative or discrete character (e.g., Warren and Mackenzie, 2001; Streisfeld and Kohn, 2005; Dick et al., 2011), here we show comparable results as using a quantitative approach. The tissue-specific regulation of the ABP (Davies et al., 2012; Albert et al., 2014), may help to explain this independent accumulation of flavonoids in *S. littorea* in different organs. The independent accumulation of flavonoids may have adaptive advantages for *S*. *littorea*. For example, the leaves and calyxes could accumulate anthocyanins or other flavonoids in response to biotic or abiotic stressors (Gould and Lister, 2006) without changing the color of the petals (Schwinn et al., 2006; Wang et al., 2013).

Conversely, when the mean population content of anthocyanins was analyzed, a positive correlation among all plant organs was found. In the pink flowered *S. dioica*, anthocyanin accumulation in petals, calyxes, and vegetative organs is greatly increased when understory plants are exposed to full sun (Kamsteeg et al., 1979). The apparent contradiction in which anthocyanin content in the different organs are unrelated at the intra-population level, but related at the inter-population level, may be explained by the lower range of climatic variation at the intra-population level than at the inter-population level, as is common in coastal species (Sagarin et al., 2006). Thus, the effect of UV-B radiation on anthocyanin accumulation is dosage-dependent (Zoratti et al., 2014). Similarly, Sperdouli and Moustakas (2012) experimentally demonstrated that anthocyanin accumulation only increased in moderate drought conditions. On the other hand, a significant correlation was only found between calyxes and leaves in the mean population content of non-anthocyanin flavonoids. This suggests that the climate has a similar effect on non-anthocyanin flavonoid accumulation in calyxes and leaves.

The relationship between the content of anthocyanins and other flavonoids in the same organ showed a well-defined pattern. At the intra-population level, the content of both groups of flavonoids was correlated in the calyx in almost all populations, and in some populations in petals and leaves. At the inter-population level, we found that both groups of flavonoids were also correlated in each of the analyzed plant parts. In genetically modified ornamental species, anthocyanin production can be modified by altering the activity of enzymes in key branches of the ABP, which generates a negative relationship between the content of flavonols and anthocyanins due to competition for a common substrate—dihydroflavonols (Holton et al., 1993; Davies et al., 2003). This competition for substrate by enzymes leading to different flavonoid branches is also suggested in *Muscari armeniacum*; plants with pigmented petals show high concentrations of anthocyanins, but low concentrations of the flavonols kaempferol and myricetin, whereas the reverse pattern was found in white-petal mutants (Lou et al., 2014). Conversely, in a comparison among petals of *Nelumbo* cultivars, a positive correlation exists between five types of anthocyanins and kaempferol derivatives (Chen et al., 2013). In *S. littorea*, our results demonstrated positive relationships between anthocyanins and other groups of flavonoids, composed of flavones and flavonols derivatives, which suggests that the sub-pathways leading to these compounds are coordinated (not competing), especially in calyxes, where positive correlations were found in almost all populations.

### Geographic and climatic variations in flavonoid content: latitude only partially explains plastic flavonoid accumulation

Considering the populations on the west coast of the Iberian Peninsula, flavonoid content in both calyxes and leaves increased toward southern latitudes. In our study, latitude greatly co-varies with mean temperature, cumulative precipitation and UV-B radiation in the growing and flowering period, as is found in studies of the same area (Narbona et al., 2010; Arista et al., 2013). Thus, increasing flavonoid contents follow a positive gradient with UV-B radiation and temperature, and a negative gradient with precipitation. Similar latitudinal variation in flavonoid accumulation with associated environmental gradients have been reported for several other plants. For instance, in northern latitudes, flavonoid content appears to increase with an increase of sunlight hours or UV-B radiation (reviewed in Jaakola and Hohtola, 2010). Higher flavonoid content has also been reported in some species in drought-stress scenarios (Chalker-Scott, 1999), but this information is based on manipulative controlled studies (e.g., Hughes et al., 2013; Ma et al., 2014), and no natural environmental gradients had previously been described before this on *S. littorea*. Conversely, *Plantago lanceolata* produced darker anthocyanin pigmented inflorescences at cooler ambient temperatures in northern populations, which increased the absorption of solar radiation and accelerated seed production (Stiles et al., 2007). Thermal acclimation is suggested to drive this variation (Lacey et al., 2010), which seems common in other species in the genus *Plantago* (Anderson et al., 2013).

The increased flavonoid accumulation associated with hotter, drier, and higher UV-B radiation populations across the western coast of the Iberian Peninsula may represent some advantages for *S. littorea*. For instance, flavonoids that accumulate in the epidermis of calyxes and leaves of *S. littorea* would mitigate the effects of solar radiation by reducing the amount of photosynthetically active radiation transmitted to chlorenchyma (Gould et al., 2010; Tattini et al., 2014) and to protect against UV-B damage to DNA (Jansen et al., 1998). Petals, which lack photosynthetic apparatus and are relatively ephemeral, would be less conditioned by such environmental gradients. In fact, the correlation between latitude and both anthocyanin and other flavonoids in petals was weak or insignificant. In addition, the reactive oxygen species‘ (ROS) scavenging capacity of flavonoids may also protect plants from drought and high temperatures common in the southern populations (Hatier and Gould, 2009; Falcone Ferreyra et al., 2012).

Unexpectedly, when all populations of *S. littorea* were considered, there was no correlation between flavonoid content and latitude. This is because the southeastern populations along the Mediterranean Sea showed very low amounts of flavonoids despite their climatic features being comparable to those of the southwestern populations (Figures 2, 5). Differences in flavonoid accumulation among populations may be caused by the combination of genetics (i.e., adaptation to local conditions) and environmental effects (i.e., phenotypic plasticity; Nicotra et al., 2010). Thus, these low values in southeastern populations would be caused by genetic constraints that restrict the adaptive potential of plants (Santamaría et al., 2003), resulting in individuals with limited capability to synthesize pigments (Jain and Gould, 2015). However, common garden experiments suggest that genetic effects are less important than environmental effects in *S. littorea* (Del Valle et al., unpublished data). Lastly, other biotic or abiotic factors (e.g., herbivory; Rolshausen and Schaefer, 2007) not considered in the present study may affect differential flavonoid accumulation in southern populations.

## Conclusions

This study accounts for a considerable variability of anthocyanins and other flavonoids in floral and vegetative organs of an annual plant species. First, the accumulation of flavonoids was highly variable among organs within individual plants. Their accumulation was independent at the population level. This within-individual variation in flavonoid accumulation may represent a component of phenotypic variability with an important adaptive value (Herrera, 2009). Interestingly, the mean population anthocyanin content in all organs was correlated, suggesting that the variable environmental conditions of coastal populations may drive anthocyanin accumulation in the whole plant. In the analysis of the populations located on the west coast, an increase of anthocyanin content in petals, calyxes and leaves, and non-anthocyanin flavonoids in calyxes and leaves was found toward southern latitudes, with higher content toward the south. The capacity to change the flavonoid accumulation in photosynthetic organs of *S. littorea* may represent an advantage for the species in climate change scenarios, where an increase of temperature and UV-B radiation is expected (Ballaré et al., 2011). Overall, this study has contributed to a more detailed understanding of how flavonoids accumulate in different plant organs, and their variation within and among populations. Despite the fact that flavonoid function is far from clearly understood (Landi et al., 2015), here we find new evidence for the relationship between anthocyanin and non-anthocyanin flavonoids in each organ, and describe a pattern of flavonoid variation along a climatic gradient. In addition, we demonstrate unexpectedly high correlations of population mean anthocyanin content between petals, calyxes and leaves that warrant further attention.

## Author contributions

EN and MB conceived of the experiments. MB, JD, JW, IC, and EN collected field data. Flavonoid extraction and quantification was performed by JD. JD, IC, and EN conducted analysis of data. JD and EN wrote the article, with assistance from all coauthors.

### Conflict of interest statement

The authors declare that the research was conducted in the absence of any commercial or financial relationships that could be construed as a potential conflict of interest.
