# Supplementary material for: On flavonoid accumulation in different plant parts: variation patterns among individuals and populations in the shore campion (Silene littorea)
Source: Front Plant Sci. 2015 Oct 29;6:939. doi: 10.3389/fpls.2015.00939 (PMC4625047; doi:10.3389/fpls.2015.00939)
Supplement: Supplementary file 1 [file Table1.DOCX]

**Supplementary Table 1. Sites, geographical locations and climatic conditions of populations of *S. littorea*.** Populations were ordered from NW to SE. For climatic data calculations, the period from February to May were used.

|  | | | | | | |
| --- | --- | --- | --- | --- | --- | --- |
| **Code** | **Locality** | **Latitude** | **Longitude** | **UV-B** | **Temp** | **Prec** |
| Bal | Balarés, A Coruña, Spain | 43º 14' 30'' N | 8º 56' 27'' W | 28.3 | 11.5 | 140.6 |
| Tre | Trece, A Coruña, Spain | 43º 10' 58'' N | 9º 09' 22'' W | 27.9 | 11.6 | 154.6 |
| Lir | Lira, A Coruña, Spain | 42º 48' 18'' N | 9º 08' 03'' W | 29.4 | 13.3 | 143.6 |
| Lou | Louro, A Coruña, Spain | 42º 46' 17'' N | 9º 07' 29'' W | 29.4 | 13.1 | 137.9 |
| Are | Areabrava, Pontevedra, Spain | 42º 17' 27'' N | 8º 50' 40'' W | 30.1 | 12.6 | 144.3 |
| Bar | Barra, Pontevedra, Spain | 42º 15' 35'' N | 8º 50' 25'' W | 30.3 | 12.6 | 138.5 |
| Mir | Miramar, Aveiro, Portugal | 41º 04' 11'' N | 8º 39' 24'' W | 32.3 | 13.3 | 103.4 |
| Naz | Nazaré, Leiria, Portugal | 39º 36' 50'' N | 9º 04' 59'' W | 33.0 | 13.3 | 78.7 |
| Cas | Cascais, Lisboa, Portugal | 38º 41' 49'' N | 9º 27' 45'' W | 33.6 | 14.4 | 62.5 |
| Alc | Alcácer do Sal, Setúbal, Portugal | 38º 29' 11'' N | 8º 54' 13'' W | 32.6 | 14.9 | 63.8 |
| Sin | Sines, Setúbal, Portugal | 37º 55' 17'' N | 8º 48' 17'' W | 34.8 | 15.1 | 63.3 |
| Alj | Aljezur, Faro, Portugal | 37º 20' 22'' N | 8º 51' 07'' W | 36.5 | 15.0 | 75.6 |
| San | Cabo San Vicente, Faro, Portugal | 37º 01' 23'' N | 8º 59' 43'' W | 36.6 | 14.5 | 67.3 |
| Odi | Odiel, Huelva, Spain | 37º 09' 14'' N | 6º 54' 19'' W | 35.2 | 15.6 | 40.4 |
| Tra | Trafalgar, Cádiz, Spain | 36º 10' 57'' N | 6º 02' 21'' W | 36.3 | 15.8 | 60.9 |
| Bre | Breña, Cádiz, Spain | 36º 11' 22'' N | 5º 56' 58'' W | 36.0 | 14.9 | 58.3 |
| Man | Manilva, Málaga, Spain | 36º 19' 57'' N | 5º 14' 21'' W | 34.5 | 15.7 | 74.1 |
| Car | Carboneras, Almería, Spain | 36º 57' 45'' N | 1º 53' 59'' W | 35.3 | 16.3 | 31.3 |
| UV-B, UV-B radiation (kJ/m^2^); Temp, mean temperature (ºC); Prec, cumulative precipitation (mm). | | | | | | |
