# Supplementary material for: On flavonoid accumulation in different plant parts: variation patterns among individuals and populations in the shore campion (Silene littorea)
Source: Front Plant Sci. 2015 Oct 29;6:939. doi: 10.3389/fpls.2015.00939 (PMC4625047; doi:10.3389/fpls.2015.00939)
Supplement: Supplementary file 2 [file Table2.DOCX]

**Supplementary Table 2.** **Summary of PCA results.** Factor loadings of climatic and geographic variables are shown.

|  | **Component 1** | **Component 2** |
| --- | --- | --- |
| Proportion of Variance  (Cumulative proportion) | 83.4%  (83.4%) | 12.4%  (95.8%) |
| Latitude | -0.977 | 0.139 |
| Longitude | 0.693 | 0.718 |
| Cumulative precipitation | -0.956 | 0.132 |
| Mean temperature | 0.966 | 0.009 |
| UV-B radiation | 0.943 | -0.259 |
