# Supplementary material for: On flavonoid accumulation in different plant parts: variation patterns among individuals and populations in the shore campion (Silene littorea)
Source: Front Plant Sci. 2015 Oct 29;6:939. doi: 10.3389/fpls.2015.00939 (PMC4625047; doi:10.3389/fpls.2015.00939)
Supplement: Supplementary file 3 [file Table3.DOCX]

**Supplementary Table 3.** **Summary of Mantel test applied to all populations and populations on the west coast of the distribution area.** Mantel’s correlation *r* is shown.

|  | **Anthocyanins** | | |  | **Non-anthocyanin flavonoids** | | |
| --- | --- | --- | --- | --- | --- | --- | --- |
|  | **Petal** | **Calyx** | **Leaf** |  | **Petal** | **Calyx** | **Leaf** |
| All populations | 0.028 | 0.002 | -0.021 |  | **0.071*** | 0.030 | -0.005 |
| W coast populations | **0.273***** | **0.244***** | **0.264***** |  | **0.046*** | **0.344***** | **0.153***** |
| Significant autocorrelations were highlighted in bold. * P < 0.05, *** P < 0.0001. | | | | | | | |
